# Gapless, Unambiguous Genome Sequence for Escherichia coli C, a Workhorse of Industrial Biology

**DOI:** 10.1128/MRA.00890-18

**Published:** 2018-10-04

**Authors:** Jonathan E. Pekar, Patrick Phaneuf, Richard Szubin, Bernhard Palsson, Adam Feist, Jonathan M. Monk

**Affiliations:** a Bioengineering Department, University of California San Diego, La Jolla, California, USA; Loyola University Chicago

## Abstract

Escherichia coli C is a commonly used strain in the bioprocessing industry, but despite its utility, the publicly available sequence of the E. coli C genome has gaps and 4,180 ambiguous base calls. Here, we present an updated, high-quality, unambiguous genome sequence with no assembly gaps.

## ANNOUNCEMENT

Escherichia coli C, an often-used industrial strain, was originally isolated by Ferdinand Hueppe from soured cow's milk and described in his 1884 publication in German (1, 2). The C strain was used extensively by Bertani and collaborators in studies of phage P2 and by many others as a strain lacking a type I restriction-modification system (3). The strain was originally termed NCTC 122 at the National Collection of Type Cultures in London, United Kingdom, where its entry states that it was deposited by the Lister Institute (London) in 1920 as “Escherichia coli” (4). This strain was recently featured in a publication comparing seven commonly used E. coli platform strains and was shown to have high anaerobic growth rates and predicted to have high relative production potential for propanol, butanol, and 3-hydroxypropanoate under anaerobic conditions (5). A draft genome sequence was deposited in the NCBI in 2016 with the GenBank accession no. MNKV00000000. This assembly has 4,180 ambiguous base calls and chromosomal gaps ranging from 45 to 125,000 bp, with a mean gap size of 7,920 bp. While this genome has already been beneficial in multistrain reconstruction work (5), analyses reliant on a pristine and accurate reference genome (e.g., single nucleotide polymorphism studies, analyses of repeat regions, etc.) are hindered by ambiguities and gaps. We therefore sequenced this strain by utilizing PacBio single-molecule and Illumina short-read sequencing and assembled the reads with Unicycler (version 0.4.2) (6). This produced an unambiguous genome sequence with no assembly gaps and an updated genome annotation.

We obtained the E. coli C strain from the DSMZ. This strain also goes by the name Sinshelmer C (DSMZ 4860, ATCC 13706, NCIB 10544). Cells were cultured overnight and DNA was extracted from the cultures using the QIAamp DNA minikit (Qiagen), as described by Monk et al. (5). Genomic DNA was prepared for PacBio and Illumina sequencing. PacBio libraries were prepared according to standard library preparation using Pacific Biosciences SMRTbell template preparation reagent kits. Libraries were size selected to >10 kb using PippinHT (Sage Sciences) and then sequenced on a PacBio RS II sequencer at the UCSD IGM Genomics Center in La Jolla, California. Illumina libraries were generated using the TruSeq DNA sample preparation kit (Illumina, Inc., USA). The libraries were sequenced using the Illumina MiSeq platform with a paired-end protocol and read lengths of 150 nucleotides (nt).

The updated C genome consists solely of a 4,614,215-bp chromosome, whereas the previous C assembly consisted of a 4,528,245-bp chromosome. This gapless assembly eliminates 4,180 ambiguous base calls compared to the previous assembly. The final assembled genome was annotated using Prokka (version 1.12) (7). The updated genome has 4,284 annotated coding sequences (CDSs) and 87 tRNAs compared with the 4,205 CDSs and 71 tRNAs in the reference annotation mentioned above. Both annotations have 8 rRNAs.

### Data availability.

This whole-genome project has been deposited in GenBank under the accession no. CP029371, and the Illumina short-read data and PacBio long-read data have been deposited in the SRA under the accession no. SAMN09199078.
